# A Study on Self-Compassion and Attachment Styles as the Predictors of Life Satisfaction in Children in Need of Protection

**DOI:** 10.3390/children12030285

**Published:** 2025-02-26

**Authors:** Elif Tuğçe Atalar, Fatih Koca

**Affiliations:** 1 Turkish Ministry of Education, Istanbul 34887, Turkey; eliftugce_yildirim20@trabzon.edu.tr; 2 Guidance and Psychological Counseling Program, Department of Educational Sciences, Graduate Education Institute, Trabzon University, Trabzon 61300, Turkey

**Keywords:** child in need of protection, life satisfaction, self-compassion, attachment styles

## Abstract

*Background and Objectives:* Societies are obliged to ensure that children in need of protection grow up psychologically, socially, and physically healthy. To achieve that, various practices have been developed. One of these practices is the use of children’s homes sites. Accordingly, this study examined the relationships between the life satisfaction, attachment styles, and self-compassion levels of children in need of protection staying in children’s homes sites. It aimed to determine to what extent variables such as attachment style and self-compassion predict life satisfaction. In addition, whether life satisfaction differs according to gender, age, and the status of having a visitor was examined. *Methods:* The study sample consisted of 121 children between the ages of 8 and 14 who were staying in children’s homes sites in Istanbul between March and May 2022. The Satisfaction with Life Scale, the short form of the Self-Compassion Scale, the Three-Dimensional Attachment Style Scale, and the Personal Information Form were used with the participants. The researchers used the independent samples *t*-test, multiple linear regression analysis, Pearson product moment coefficient, and one-way analysis of variance (ANOVA) during the analysis. *Results:* The findings showed that there was a significant positive relationship between secure attachment style and life satisfaction, anxious–ambivalent attachment, and self-compassion. Also, among the demographic variables, being visited by relatives was found to predict life satisfaction, while there was no relationship between life satisfaction and age and gender. *Conclusions:* The study findings were discussed within the framework of the literature, and suggestions based on the findings were presented.

## 1. Introduction

The survival of societies is possible through the healthy upbringing of the child population [1]. Childhood is a crucial developmental stage in which human life is shaped, and spending it with the family is considered essential for its healthy progress [2,3]. However, for many reasons, such as family breakdown [4,5,6], financial inadequacies [7,8,9], neglect and abuse [10], below average intelligence [11], teenage marriage, or abandonment as a result of out-of-wedlock births, families are unable to play a role during the development of their children and the children become in need of protection [3]. A child in need of protection is defined by Article 3 (b) of the Social Services Law No. 2828 of Türkiye, published in Türkiye’s Official Gazette No. 18059 dated 27 May 1983, as follows:

A child in need of protection is defined as a child whose physical, mental, and moral development or personal safety are in danger; who is without a mother or father; whose mother or father or both parents are unknown; who has been abandoned or neglected by his/her mother or father or both parents; who has been left defenseless against all social dangers and harmful habits, such as prostitution, begging, and alcohol or drug use; and who falls into vagrancy.

States are obliged to take measures in line with their own standards for the protection of children whose development, education, and care have been disrupted, regardless of the cause [12]. Practices developed for the care of children in need of protection vary across countries [13]. In Türkiye, the studies on child welfare are based on existing legal regulations and social policies. In line with these regulations and policies, the state provides various services for the care and protection of children through its institutions. Currently, the institution undertaking this task is the General Directorate of Child Services under the Ministry of Family and Social Services of Türkiye.

In the past, children were provided with care and protection in barracks-type institutions, where too many children lived together. Subsequently, studies showed that barracks-type institutions caused negativities in children’s development by making it difficult to provide care sensitive to their individual differences [2,3,14,15,16,17]. Changed to support the healthy development of children, barracks-type institutions were thereupon replaced by institutions physically resembling the home environment, where fewer children live together. In Western countries with developed industries, institutions where many children live together have been closed for more than 30 years, while Asian and African countries do not have enough resources to make this change fully [18,19]. Although it is clear that the ideal is to completely eliminate institutional care, it is difficult to find quality family care for children affected by wars and disasters, homeless children, and those who are mistreated by their families. Therefore, the most viable option is to maintain children’s homes that are closest to the family environment, and it is crucial that these environments have more favorable opportunities than the environments that the children come from [20]. Wolf and Fesseha defined the ideal environment for children in need of protection and care as SOS Children’s Villages, consisting of houses with 6–8 children where 24/7 continuous care is provided by the same caregiver [19]. Similarly, the care service in Türkiye provides children’s homes sites. Children’s homes sites are included in the Social Services Board’s decision no. 2828. These homes are a care service where 10–12 children of the same sex live together, with a relationship network and structure similar to that of the family environment, consisting of low-rise houses within a well-defined housing estate. Based on the goal of ensuring the formation of a basic sense of trust in children, a continuous care service with a small number of unchanging personnel is aimed at [21]. In Türkiye, a considerable number of children in need of protection benefit from this service. The latest statistical data, published by the Ministry in 2022, show that there are 6669 children in care in 113 homes [22]. Research on children in need of protection, who constitute a significant part of the child population in Türkiye, is limited. Moreover, no studies have examined the study variables together in this specific sample. Therefore, children in need of protection were chosen as the target group for this study.

According to Freud [23], in order for a child to complete the developmental stages healthily, the attention and affection received from the mother is of great importance, and no other place can replace the love and care of the family. From the moment an individual is born, survival and happiness are prominent as his/her basic needs [24]. Life satisfaction is defined as the result obtained by an individual after comparing what s/he expects from life with what s/he already has [25]. While there are many studies on adults’ life satisfaction, there has been an increase in the number of studies on the life satisfaction of children and adolescents recently [26]. However, studies on the life satisfaction of children in need of protection are limited. Studies on children and adolescents show that high life satisfaction enables the development of positive behaviors in relevant samples, leads to an increase in their happiness, and encourages them to explore life [27,28,29]. Elattar, Alabd, and Mohammed demonstrate in their study that children deprived of family love and attention are more prone to behavioral and emotional problems than their peers and that these problems affect their life satisfaction [30].

The literature reveals that there are various factors affecting satisfaction with life. These factors can be listed as physical health, economic security, social relationships, satisfaction with the institution where the care is received, physical activity, and incidents encountered in life [31,32,33,34]. In addition, some demographic variables are known to affect satisfaction with life. A meta-analysis of 46 studies on the relationship between life satisfaction and gender found that there is no significant relationship between gender and life satisfaction, but male children and adolescents have relatively high life satisfaction [35]. In the studies on the age variable, different results have been encountered. Resembling a U shape, life satisfaction is stated to decrease toward middle age and then increase with age [36,37]. However, recent studies reveal that there is no relationship between age and life satisfaction [38,39,40,41,42,43]. The results on different samples may depend on the samples’ characteristics. Thus, the relationship between life satisfaction and age was analyzed in our study. The other variable analyzed was the status of children in children’s homes who are visited by their families. Children staying in children’s homes can be visited by their families. After her study on the behavioral problems of children in orphanages, Saraç [44] found that being visited has a good long-term effect on the children’s mental health. Based on this result, the relationship between being visited and life satisfaction levels was analyzed.

Attachment develops and is shaped by the interaction between the infant and the caregiver, starting from the prenatal period [45]. Children who have experienced relationship trauma at an early age have difficulty establishing a harmonious relationship and connection with their caregivers [46]. Children separated from their families for certain reasons and put into institutional care have been observed to have lower secure attachment styles and higher levels of disorganized attachment disorder than those living with their parents [47,48,49,50,51,52]. Individuals who have developed a secure attachment with their caregivers at an early age can develop healthily [53], establish strong relationships in adolescence and adulthood [54], and cope with negative emotions more easily [55]. In addition, individuals with secure attachment styles are known to perceive themselves positively and be perceived positively by other people [56]. These findings reveal the important effect of secure attachment that individuals form in childhood on their future lives.

Self-compassion, another dependent variable of this study, means that individuals are aware of their own pain, accept that negative experiences are experiences that can happen to everyone, and create a desire to heal themselves by approaching themselves with kindness [57]. The social environment in which children live has a great role in the development of self-compassion. Individuals who are not approached compassionately by their caregiver in their early life, whose needs are not met, and who are not soothed cannot form brain connections about these experiences. As a result, it is expected that they cannot show self-compassion [58,59]. It is emphasized that family is a crucial factor in the development of early maladaptive schemas [60]. Similarly, Neff (2011) stated that a parent’s approach to his/her child at an early age affects the child’s self-behaviors throughout life and that the level of self-compassion is related to early childhood experiences [61]. On the other hand, Cohen and Wills (1985) suggested that social support protects individuals from the damaging effects of stress during difficult life events by acting as a buffer and helps them maintain their mental and physical health [62]. Accordingly, self-compassion supports individuals against negative life events and is a healthy way to cope with these experiences [57,63]. Studies have also found significant positive relationships between self-compassion and life satisfaction [64,65,66]. Children in need of protection are those who have faced challenging life events at an early age and live away from their families, who are a critical element of social support. Conducted in this context, a study found that children staying in such institutions have low levels of self-compassion [67]. Similarly, this study aims to examine the predictive power of self-compassion levels on life satisfaction in the target group.

Based on the literature results, this study aims to examine self-compassion and attachment styles as predictors of the life satisfaction of children in institutional care and to reveal if life satisfaction differs according to gender, age, and status of being visited. In this context, the life satisfaction of individuals staying in children’s homes may contribute to them being psychologically healthy individuals in the future and indirectly to social development. Accordingly, the results obtained regarding the variables may guide the planning of preventive and interventional studies to increase the life satisfaction of children in need of protection. Through this study, assessing self-compassion and attachment styles as predictors of the life satisfaction of children in need of protection may shed light on the work areas of psychological counselors, psychologists, social workers, and various other professional groups who work directly with children staying in these institutions.

The main purpose of this study is to examine how attachment styles and self-compassion levels in children in need of protection predict their life satisfaction and to see the relationships between these variables. Additionally, the study aims to investigate how these variables vary based on demographic variables (gender, age, and whether the children are visited by their families).

In line with this purpose, the hypotheses of the study are as follows:

1. Self-compassion and attachment styles in children in need of protection predict their life satisfaction.
2. Self-compassion, attachment styles, and life satisfaction in children in need of protection differ significantly based on gender, age, and whether they are visited by their families.

## 2. Materials and Methods

### 2.1. Study Group

The study group consisted of 121 children between the ages of 8 and 14 staying in Küçükyalı Children’s Homes Site Directorate, Yakacık Hatice Abbas Halim Children’s Homes Site, Beykoz Galip Öztürk Children’s Homes Site, and Bahçelievler Şeyh Zayed Children’s Homes Site in Istanbul between March and May 2022. Of the participants, 54 were girls and 67 were boys. The group was determined through convenience sampling, a non-random sampling method. Table 1 shows the detailed demographic information of the participants.

### 2.2. Process

Firstly, ethics committee permission was obtained from Trabzon University Social and Humanities Scientific Research and Publication Ethics Committee (dated 24 September 2021 and numbered E-81614018-000-788). The necessary permissions to use the measurement tools in the children’s homes sites were obtained from the Directorate of Training and Publication, Ministry of Family and Social Services of Türkiye. After all the permissions were received, the children’s homes sites in Istanbul were contacted and checked to see if they were suitable for data collection. Among the sites contacted, Küçükyalı Children’s Homes Site Directorate, Yakacık Hatice Abbas Halim Children’s Homes Site, Beykoz Galip Öztürk Children’s Homes Site, and Bahçelievler Şeyh Zayed Children’s Homes Site were determined to be suitable, and a date and time were assigned for the data collection. Copies of the forms were produced and used over a period of 2 months, starting from March 2022. Before starting the form, the staff were given an explanation of the Personal Information Form to be filled out by them. The children whose personal information forms were filled out were included in the study after they were also given the relevant explanation. Those between the ages of 8 and 14 who could not read and write were not included in this study.

### 2.3. Data Collection Tools

The Satisfaction with Life Scale: Developed by Diener et al. to determine the level of satisfaction with life [68], the scale was adapted into Turkish by Dağlı and Baysal [69]. It is a 5-point Likert-type scale with no reverse items. For its internal consistency, the Cronbach alpha value was found to be 0.88, which shows that the internal consistency is high. In addition, its test–retest reliability was measured as 0.97. The factor analysis demonstrated that the Turkish version of the Satisfaction with Life Scale consists of five items with a single dimension, similar to the original one. The items are as follows: “1. I have a life close to my ideals in many aspects. 2. My living conditions are perfect. 3. My life satisfies me. 4. So far, I have achieved the important things I want in life. 5. If I had the chance to live my life again, I would change almost nothing” [69].

The Self-Compassion Scale—Short Form: The Self-Compassion Scale was developed by Neff [57] and adapted to its short form by Raes, Pommier, Neff, and Van Gucht [70]. Through a validity and reliability study, it was adapted to Turkish by Yıldırım and Sarı (2018). Its Cronbach alpha internal consistency coefficient is 0.75, and it is a 5-point Likert-type scale with 11 questions. In the scale, six items, namely items 1, 4, 8, 9, 20, and 11, are reverse scored. In the scale, which consists of a single dimension, the statements are scored between “never (1)” and “always (5)”. A minimum of 11 and a maximum of 55 points can be obtained from it. A high score indicates a high level of self-compassion [71].

The Three-Dimensional Attachment Style Scale: The scale was developed by Erzen [72] to reveal attachment styles, including secure attachment, anxious–ambivalent attachment, and avoidant attachment. It has 18 items in a 5-point Likert-type scale. The items are scored from “strongly disagree (1)” to “strongly agree (5)”. The scale consists of two negative (anxious–ambivalent and avoidant) and one positive (secure) attachment sub-dimensions. The Cronbach alpha internal consistency coefficient was calculated as 0.69 for the secure attachment sub-dimension, 0.71 for the anxious–ambivalent attachment sub-dimension, and 0.80 for the avoidant attachment sub-dimension. As the exploratory factor analysis revealed, 18 items in the scale are consistently clustered under the three dimensions. The item–total correlation values range between 0.49 and 0.75.

The Personal Information Form: Created by the researcher through a literature review, the form has descriptive information about the children in need of protection. It includes closed-ended questions such as the child’s age, length of stay in the institution, the reason for admission to the institution, the number of siblings, the status of being visited by the family, and school attendance. The relevant scale was filled out by the member of staff who is the child’s personal caregiver.

### 2.4. Data Analysis

The SPSS v29.01.0 statistical package program was used to analyze the study data. The completed forms were checked to see if there were any incomplete or incorrect ones among them, and nine forms that were found to be incorrectly completed were not included in the evaluation. Then, one-way extreme values were calculated. All items were converted into standard z scores, and one-way outliers were checked. Tabachinck and Fidell [73] define outliers as those with z scores that are outside the range of −3.29 to +3.29. Thus, no outlier outside the relevant range was found in the data. Next, the normality of the distribution was examined through the data’s kurtosis and skewness coefficients. According to Tabachnick and Fidell [73], a normal distribution is possible when all values are between −1 and +1. Therefore, the data were seen to be suitable for normal distribution. The mean, standard deviation, kurtosis, and skewness coefficients and the minimum and maximum scores given by the participants in the forms were calculated using descriptive statistical techniques. Table 2 shows the related values.

To use the regression analysis method in this study, the correlation between the independent variables should be 0.80 or lower [74]. Thus, to determine if this assumption was met, the relationship between the mean scores of each scale in the study was assessed through Pearson correlation. An evaluation of the values in Table 3 showed that the assumption required for the use of the regression analysis method was met.

After analyzing the correlation values between the variables, Levene’s F test was applied to test the homogeneity of variance, showing that the study data are homogeneously distributed. Thus, parametric methods were preferred in all the analyses. The assumption for each method was assessed based on a significance level of 0.05. An independent samples *t*-test was used for the difference in the participants’ life satisfaction scores according to gender and the status of being visited, ANOVA was utilized for the difference in the life satisfaction scores according to age, and multiple linear regression was used to see whether self-compassion and attachment styles predict the life satisfaction scores.

## 3. Results

Table 4 shows the results of the multiple linear regression applied to test if attachment styles and self-compassion can predict satisfaction with life.

The results of the regression model applied to determine the effect of the attachment style sub-dimensions and self-compassion levels on the life satisfaction levels of the children in need of protection are statistically significant (F(5.445) = 45.401, *p* = 0.021, *p* < 0.05). Adjusted R^2^ indicates how much of the change in the dependent variable is explained by the independent variables [75]. Thus, 7.5% of the life satisfaction scores of the children in need of protection are explained by the attachment styles scores.

Among the model’s independent variables, the secure attachment sub-dimension of attachment styles significantly predicts the children’s life satisfaction (B = 0.323, t = 2.909, *p* < 0.05). The secure attachment sub-dimension positively affects their life satisfaction. However, the avoidant (B = −0.013, t = −0.146, *p* > 0.05) and anxious (B = −0.149, t = −1.507, *p* > 0.05) sub-dimensions of the attachment styles do not significantly predict their life satisfaction. In the model, the independent variable of self-compassion does not significantly predict their life satisfaction (B = 0.093, t = 1.112, *p* > 0.05).

In this study, the independent samples *t*-test was used to determine if the participants’ life satisfaction differs according to gender. There is no statistically significant difference between the life satisfaction scores according to gender (t(119) = 1.046, *p* > 0.05). Please see Table 5.

In this study, the independent samples *t*-test was used to determine if the participants’ life satisfaction differs according to the status of being visited. There is a statistically significant difference between the life satisfaction scores according to the status of being visited (t(119) = 1.780, *p* < 0.05). Please see Table 6.

In this study, one-way analysis of variance (ANOVA) was used to determine if the participants’ life satisfaction differs according to age. Table 7 and Table 8 show these data.

As shown in the table, the life satisfaction score means of the children differ from each other according to their ages. Table 7 shows that the group with the highest life satisfaction score mean is that of the 8–10-year-old children, and the group with the lowest life satisfaction score mean is that of the 10–12-year-old children. ANOVA was applied to determine if the difference in these score means is statistically significant. The analysis findings are shown in Table 8.

Table 8 shows that there is no significant difference between the life satisfaction score means of the children according to age (F(2,119) = 2.62, *p* > 0.05).

## 4. Discussion and Conclusions

In this study, attachment styles and self-compassion were examined as predictors of life satisfaction. The findings show that secure attachment, one of the attachment styles, significantly predicts life satisfaction positively, while no relationship was found between the anxious and avoidant attachment styles and life satisfaction.

A study by Ünal and Şahin [43] on high school children found that secure attachment predicts life satisfaction positively and significantly, while anxious and avoidant attachment styles do not predict life satisfaction significantly. There are also other studies in the literature that found that secure attachment predicts life satisfaction positively [76,77,78,79]. These are consistent with our study’s findings. However, in his study, Gürçam did not find any relationship between attachment styles and life satisfaction [80]. These different results on the relationship between life satisfaction and attachment styles may be related to the difference in the study groups and the presence of confounding variables.

In his attachment theory, Bowlby stated that secure attachment occurs when infants’ needs are met with a trust-based relationship [81]. A study showed that the caregiver’s personality traits, commitment to the child, and care for the child’s needs are highly likely to develop a secure attachment style [82]. In this regard, children in need of protection who have a secure attachment style may have previously established a trust-based relationship with a caregiver. In addition, although attachment styles acquired at an early age are known to be transferred to future periods and generalized, studies demonstrate that individuals with insecure attachment styles may experience changes in their attachment schemas when they are provided with the love and support that they need [83]. Children who were exposed to negative experiences in infancy and could not develop a secure attachment style may have changed their attachment schemas by being provided with support, such as continuous and unchanging care and one-to-one relationships, under state protection, and accordingly, their life satisfaction level may have increased. Considering that secure attachment is formed as a result of children’s unchanging and continuous bond with their caregiver, ensuring the continuity and unchangeability of the bond between caregivers and children living in institutional care is thought to be effective in creating secure attachment. Also, psycho-educational interventions can be organized for caregivers who interact with children in children’s homes to ensure secure attachment with the children they are responsible for.

Parade, Leerkes, and Blankson found that secure attachment facilitates establishing a relationship with friends and participating in social life [84]. Individuals who participate in social life and make friends are expected to engage in more activities. According to activity theory, one of the theories explaining life satisfaction, happiness is the result of the activities performed by individuals [68]. Similarly, studies have found that leisure time activities increase individuals’ strength to cope with negativities and improve their life satisfaction [85,86]. In view of this, we can conclude that secure attachment increases participation in activities, which affects life satisfaction. Likewise, Perrone, Webb, and Jackson stated that the life satisfaction factor is affected by experiences, such as environmental conditions and attachment in relationships [87].

Rothbard and Shaver concluded that individuals with a secure attachment style experience less negative affect, establish healthier relationships, and are more sensitive to the problems of the people around them [88]. Neugarten, Havighurst, and Tobin identified having positive affect in the face of life events as one of the five criteria to determine life satisfaction [25]. In this context, it can be inferred that having a secure attachment style leads to positive affect, and this may predict life satisfaction.

In this study, no relationship was found between anxious and avoidant attachment styles and life satisfaction, which is the dependent variable of the study. Studies on the attachment styles of children in institutional care reveal that among the attachment styles, disorganized attachment is often prominent [48,51,52]. However, the fact that there is no study in the literature directly examining the relationship between attachment styles and life satisfaction in a relevant sample makes it difficult to comment on these findings. The expected result of the study is that avoidant and anxious attachment styles are related to life satisfaction negatively. Various studies with such findings are found in the literature [89,90]. However, the sample of children in need of protection is a limited sample that is difficult to contact. This limitation regarding the study sample may have prevented the effect of anxious and avoidant attachment sub-dimensions’ predictivity.

Multiple studies point out that traumatic experiences significantly and negatively affect life satisfaction [91]. However, trauma may not always have negative effects. In this regard, the notion of “posttraumatic growth” has recently come to the fore, and there are many studies on this notion [92,93,94,95]. Posttraumatic growth is defined as positive changes in individuals’ relationships, outlook on life, and self-evaluations after a life crisis or trauma [96]. Veronese and Pepe showed that child victims of war become resistant to posttraumatic stress disorder and adapt to trauma [97]. In light of this, it is probable that children who have been placed under state protection after going through traumatic experiences have changed and transformed by adapting to the situation they live in and have not developed avoidant and anxious attachment patterns, and thus, anxious and avoidant attachment styles have not entered the model as a negative predictor of life satisfaction.

As a result, the findings and the literature show that the relationship and support that children in need of protection establish with their caregiver in the place where the care is given will positively affect their life and prepare them for healthy adulthood, considering the positive and significant relationship between the secure attachment style and life satisfaction. Based on these results, policymakers can minimize the number of children in institutional care by determining policies that increase the number of foster families, adoption, and family support services, which are alternative care methods that allow these children to spend more time with caregivers and bond securely with them.

Another variable whose relationship with life satisfaction was examined in this study is self-compassion. The findings show that there is no relationship between self-compassion and life satisfaction. In the literature, there are various studies on the relationship between self-compassion and life satisfaction. In her study on relationships between self-compassion and depression, self-esteem, anxiety, need satisfaction, self-acceptance, perfectionism, narcissism, and life satisfaction, Neff [57] found that self-compassion has a positive and significant relationship with self-esteem, life satisfaction, and self-acceptance. Similarly, there are studies in the literature that have found a significant positive relationship between self-compassion and life satisfaction levels [64,98]. Yet, there are few studies with a sample similar to our study’s sample. Sinta and Zulkarnain examined the self-compassion levels of adolescents in institutional care [67] and discovered in their study of 392 adolescents that the adolescents have low self-compassion levels. Aulia and Rahayu, however, found a significant positive relationship between self-compassion and life satisfaction in their study on 35 adolescents in an institution in state care [66]. All these studies reveal that self-compassion has a significant effect on life satisfaction. Contrary to these findings, no relationship was found between self-compassion and life satisfaction in our study.

Self-compassion is known to help individuals to develop positive affect in negative situations and protect themselves from negative thoughts [63,99]. Thus, self-compassion can be seen as a protective factor in tough situations for children staying in children’s homes sites. However, this sample consists of children who were generally exposed to difficult life events. Leaving their family and starting to live in a place where they have not been before is expected to be stressful and traumatic. Yet, self-compassion is affected by early life experiences. Negative experiences affect people negatively and may damage their self-perception [100]. The answers to the open-ended question “the reason why the individual was put under protection” in the Personal Information Form revealed that the majority of the participants were placed under protection and care due to traumatic experiences (abuse, neglect, breakdown of family, etc.). Likewise, Siegel stated that uncertain and uneven processes in life and the inability to have control over these processes cause uncertainty in the perception of self-compassion in individuals [101]. It is usually uncertain how long children staying in such homes will stay there. In this study, it was considered that the variable of self-compassion could not enter the model as a predictor of the participants’ life satisfaction due to living with the mentioned uncertainties and challenging experiences.

Lane and Schwartz define emotional awareness as the ability to recognize one’s own and others’ emotions [102]. In her study, Yılmazörnek [103] noticed that those with emotional deprivation, pessimism, social isolation, failure, dependency, weakness, and abandonment schemas among early maladaptive schemas have low emotional self-awareness. According to the current study’s findings, children in need of protection who have difficult experiences at an early age may be expected to have low emotional self-awareness. Low emotional awareness may prevent the self-compassion variable from having a significant effect on life satisfaction.

This study examines whether the life satisfaction of children in need of protection differs in terms of gender, frequency of being visited, and age variables. Firstly, no significant relationship was found between gender and life satisfaction in the study. The literature shows that there are various findings on the relevant subject. In their study, Grant, Wardle, and Steptoe found that the life satisfaction of boys is higher than that of girls [104]. Likewise, there are other studies showing that boys have higher life satisfaction than girls [105,106]. Yet, there are also studies noting that the life satisfaction level of girls is significantly higher than that of boys [107,108]. Similar to our study sample, a study by Korkmaz of 111 children in institutional care found no relationship between life satisfaction and gender [33]. In the literature, there are similar studies with no relationship identified between gender and life satisfaction [109,110,111,112,113,114]. These findings, which are not sufficiently compatible with each other, suggest that the effect of gender on life satisfaction may differ according to the characteristics of the society or the sample. Statistically insignificant findings may become significant as the sample size increases [115]. Also, the sample is a special sample that is difficult to reach. Therefore, the number of participants is relatively small. The limitations related to the sample and the number of participants have created difficulties in testing the study hypotheses.

The family’s role in the development of children’s gender perceptions is crucial. Families expect children to show behaviors appropriate to their gender and direct them this way [116]. However, children in children’s homes sites live in groups of 6–8 people, away from parents who they can take as role models and from a real family environment. Although there is a caregiver mother/father practice in these homes, it is likely that they cannot provide a real home experience. Thus, the lack of a significant effect of the gender factor on life satisfaction in this study is considered understandable.

In their study, Hilliard and Liben found that as children spend longer in groups of friends with gender-based discrimination, their gender stereotypes increase [117], they make fewer positive judgments toward their opposite-sex peers, and they want to play less with them, while children in groups without gender-based discrimination do not have such tendencies. As it is known, children’s homes sites are institutions where children of the same sex live together. In this regard, gender-based discrimination may have led to an increase in the children’s stereotypes, and this may be the reason why a significant relationship is not found between their life satisfaction and the variable of gender.

The second variable whose relationship with life satisfaction was analyzed in the study is the frequency of being visited. A significant relationship was found between life satisfaction and the variable of being visited. Accordingly, the life satisfaction levels of children being visited are higher than those of children not being visited. In the literature, there is no study specifically on the relationship between life satisfaction and the status of being visited.

Social support is a feeling of being valued and seen as important by others that a person cares about in his/her life [118]. The study by Öğütcü on children in Child Support Centers found a high perception of social support among those being visited by their relatives [119]. In their study, Topkaya and Büyükgöze-Kavas revealed that perceived social support has a predictive role in life satisfaction and that individuals with high perceived social support have high life satisfaction [120]. Therefore, the social support perceived by the children who are being visited is considered to be high, and as a result, their life satisfaction is high. In this context, examining the mediating role of perceived social support might be important for elaborating on this subject in future studies.

The Child Protection System Evaluation Report of SHÇEK (Social Service and Children Protection Agency of Türkiye) shows that less than 17% of parents visit their children in institutional care often [121]. However, being visited in institutional care is crucial for the children’s psychosocial development. If not visited, they may feel orphaned and abandoned, retreat into their shells, break their ties with their environment, and develop antisocial personalities [6]. In her study on children in children’s homes, Yorgun found that being visited by relatives positively affects the children’s moral value structures [122]. According to Yurdakul, the frequency of the mother’s visits is effective and important for the child’s psychosocial development [123]. Therefore, being visited may lead the children to feel positive emotions while evaluating their lives, and thus their life satisfaction levels become high. Based on the fact that being visited increases children’s life satisfaction, as found in this study, families can be informed about its importance and encourage them to visit their children. However, the fact that this study is not a causal one prevents us from reaching a definite conclusion. In future studies with a causal or mixed design, the relationship between the status of being visited and life satisfaction can be examined in depth.

Another variable whose relationship with life satisfaction was analyzed in the study is age. This study discovered that there is no significant relationship between age and life satisfaction. In the literature, there are many studies on the relationship between life satisfaction and age, and there are different opinions on this subject. In their study on children and adolescents in Zambia, Holder, Coleman, T. Krupa, and E. Krupa discovered that age has a weak predictivity on life satisfaction [124]. As a result of their study, Chang, McBride-Chang, Stewart, and Au found that life satisfaction, which is relatively high in childhood, decreases in the first years of adolescence [125]. Likewise, a study on the developmental dimensions of life satisfaction in children aged 11–16 years showed that life satisfaction decreases in later ages [105]. Yet, in a study with a sample of children in need of protection, Korkmaz found no relationship between life satisfaction levels and age, which is consistent with our findings [33]. These contradictory findings show that more research is needed in this field. According to Diener, subjective well-being is more strongly related to socioeconomic status than age and gender and is perhaps its strongest socio-structural predictor [126]. Thus, age may not have enough influence to create a significant difference in determining the life satisfaction of the children with the same living conditions in children’s homes sites.

## 5. Limitations

There are some limitations to this study. It was conducted within a limited period using quantitative research methods. In addition to the scales used, qualitative research methods can be utilized to explore the subject in greater depth. Additionally, the study is limited to children residing in children’s homes sites in the city of Istanbul. This sample is a group that is challenging to reach and collect data from. Various lengthy legal procedures are required to reach the relevant group. Consequently, the number of participants in the study was limited to 121.

## Figures and Tables

**Table 1:** Demographic information of the participants.

Variables	Group	Frequency (N)	Percentage (%)
Gender	Female	54	44.62
	Male	67	55.37
Age	8–10	38	31.40
	10–12	39	32.23
	12–14	44	36.36
Number of Siblings	No Sibling	22	18.18
	1 Sibling	26	21.48
	2 Siblings	45	37.19
	3+ Siblings	28	23.14
Duration of Stay in the Institution	Less than 1 year	37	30.57
	1 year	27	23.31
	2 years	16	13.22
	More than 2 years	41	33.88
Continuing His/Her Education	Continuing	118	97.52
	Not Continuing	3	2.47
Status of Being Visited	Being Visited	64	52.89
	Not Being Visited	57	47.10
Total		121	100

**Table 2:** Descriptive statistics results regarding the variables.

Variable	N	Min.	Max.	X	SD	Skewness	Kurtosis
Satisfaction with Life	121	5	25	13.70	0.494	0.280	−0.716
Self-Compassion	121	5	46	33.12	0.573	−0.438	0.414
Secure Attachment	121	6	25	17.68	0.415	−0.252	−0.519
Avoidant Attachment	121	7	33	16.79	0.533	0.304	−0.355
Anxious Attachment	121	7	30	17.29	0.493	0.257	−0.702

X = mean, Sd = standard deviation, Min = minimum, Max = maximum.

**Table 3:** Table of correlation between the variables.

	X	Sd	1	2	3	4	5
1. Satisfaction with life	13.70	5.45	1	0.10	0.29	−0.11	−0.09
2. Self-Compassion	33.12	6.30	0.10	1	0.20 *	0.11	0.37 **
3. Secure Attachment	17.68	17.68	0.29	0.20 *	1	−0.24	0.05
4. Avoidant Attachment	16.79	16.79	−0.11	0.11	−0.24 **	1	0.29 **
5. Anxious Attachment	17.29	17.29	−0.09	0.37	0.05	0.29	1

* *p* < 0.05, ** *p* < 0.01. X = mean, Sd = standard deviation.

**Table 4:** Multiple linear regression results of life satisfaction score means according to attachment styles and self-compassion.

Model		B	Standard Error	ß	T	*p*
1	Fixed	7.696	3.277		2.348	0.021
	Secure Attachment	0.323	0.109	0.272	2.909	0.004
	Avoidant Attachment	−0.013	0.089	−0.014	−0.146	0.884
	Anxious Attachment	−0.149	0.099	−0.148	−1.507	0.135
	Self-Compassion	0.093	0.084	0.108	1.112	0.269
R = 0.326	R^2^ = 0.106		Adjusted R^2^ = 0.075		F(4,121) = 3.445	

**Table 5:** Independent samples *t*-test results of life satisfaction score means according to gender.

Group	N	X	SD	t	DF	*p*
Male	54	14.28	5.82	1.046	119	1.82
Female	67	13.24	5.09			

X = mean, SD = standard deviation, DF = degrees of freedom.

**Table 6:** Independent samples *t*-test results of life satisfaction score means according to the status of being visited.

Group	N	X	SD	t	DF	*p*
Being Visited	64	14.12	5.64	1.780	119	0.047
Not Being Visited	57	10.24	3.01			

X = mean, SD = standard deviation.

**Table 7:** Descriptive statistics results of life satisfaction score means according to age.

Dependent Variable	Age	N	X	SD
Life Satisfaction	8–10	38	15.08	0.804
	10–12	39	12.28	0.865
	12–14	44	13.77	0.853

**Table 8:** ANOVA results of life satisfaction score means according to age.

Source of Variance	Sum of Squares	Degrees of Freedom	Mean Square	F	*p*
Between Groups	150.90	2	75.45	2.62	0.077
Within Groups	3394.38	119	28.76		
Total	3545.28	121			

## Data Availability

The data presented in this study are available on request from the corresponding author. The data are not publicly available due to restrictions, e.g., privacy or ethical.
